# 1-(2,4-Dichloro­phen­yl)-4,4-dimethyl­pent-1-en-3-one

**DOI:** 10.1107/S1600536808029838

**Published:** 2008-09-20

**Authors:** Lin Xia, Ai-Xi Hu

**Affiliations:** a College of Chemistry and Chemical Engineering, Hunan University, 410082 Changsha, People’s Republic of China

## Abstract

In the title compound, C_13_H_14_Cl_2_O, the carbonyl and ethenyl groups are coplanar with the aromatic ring. There are four molecules in the asymmetric unit and all atoms in the molecule lie on mirror planes. The mol­ecules are packed in an offset face-to-face arrangement showing π–π stacking inter­actions involving the benzene rings [centroid–centroid distance = 3.564 (2) Å].

## Related literature

For related compounds, see: Wang *et al.* (2008 ▶).
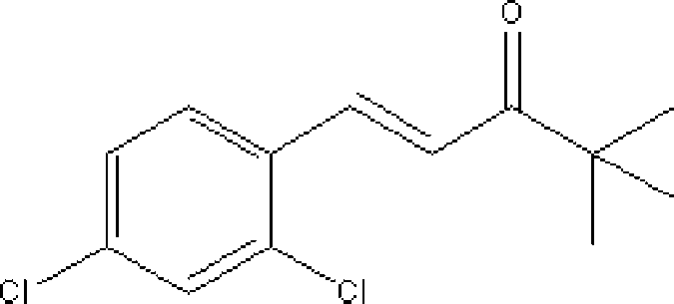


## Experimental

### 

#### Crystal data

C_13_H_14_Cl_2_O
*M* *_r_* = 257.14
Orthorhombic, 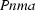

*a* = 11.2553 (7) Å
*b* = 7.0458 (4) Å
*c* = 15.4969 (9) Å
*V* = 1228.94 (13) Å^3^
*Z* = 4
Mo *K*α radiation
μ = 0.50 mm^−1^
*T* = 173 (2) K
0.47 × 0.39 × 0.15 mm

#### Data collection

Bruker SMART 1000 CCD diffractometer
Absorption correction: multi-scan (*SADABS*; Sheldrick, 2004 ▶) *T*_min_ = 0.798, *T*_max_ = 0.928
6162 measured reflections
1444 independent reflections
1227 reflections with *I* > 2σ(*I*)
*R*_int_ = 0.021

#### Refinement

*R*[*F* ^2^ > 2σ(*F* ^2^)] = 0.032
*wR*(*F* ^2^) = 0.085
*S* = 1.07
1444 reflections
95 parameters
H-atom parameters constrained
Δρ_max_ = 0.28 e Å^−3^
Δρ_min_ = −0.36 e Å^−3^

### 

Data collection: *SMART* (Bruker, 2001 ▶); cell refinement: *SAINT-Plus* (Bruker, 2003 ▶); data reduction: *SAINT-Plus*; program(s) used to solve structure: *SHELXTL* (Sheldrick, 2008 ▶); program(s) used to refine structure: *SHELXTL*; molecular graphics: *SHELXTL*; software used to prepare material for publication: *SHELXTL*.

## Supplementary Material

Crystal structure: contains datablocks global, I. DOI: 10.1107/S1600536808029838/sg2260sup1.cif

Structure factors: contains datablocks I. DOI: 10.1107/S1600536808029838/sg2260Isup2.hkl

Additional supplementary materials:  crystallographic information; 3D view; checkCIF report

## Figures and Tables

**Fig. 1.**
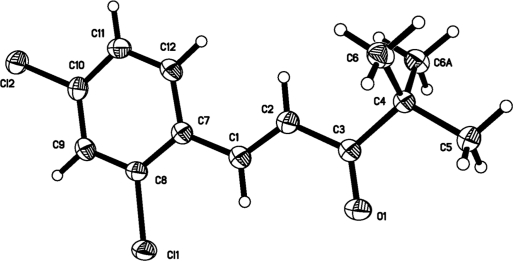
Molecular structure showing 30% probability displacement ellipsoids.H atoms are omitted for clarity.

**Fig. 2.**
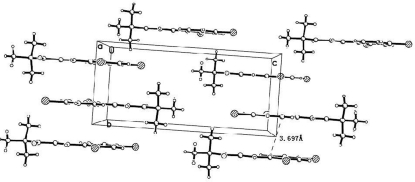
Packing diagram showing π–π stacking interactions.

## supplementary crystallographic information

### Comment

Phenyl-4,4-dimethylpentan-3-one derivatives are  important intermediates in medicinal industry (Wang *et al.*, 2006). Herein we report the synthesis and crystal structure of 1-(2,4-dichlorophen-yl)-4,4-dimethylpentan-3-one.

### Experimental

3,3-dimethylbutan-2-one(0.0105 mol) was added dropwise into a solution of 2,4-dichlorobenzaldehyde (0.01 mol) and 60 ml ethanol. Then 0.1 g 50% NaOH solution as catalyst was added and the reaction was stirred at 333 K for 5 h . The solvent was evaporated to about half volume, then cooled to 277 K and the precipitate formed.This was filtered and dried to give the desired product (yield: 94.7%). Crystals suitable for X-ray structure determination were obtained by slow evaporation of an ethanol solution at room temperature.

### Refinement

The methyl H atoms were positioned geometrically (C—H=0.98 Å) and torsion angles refined to fit the electron density [*U*_iso_(H) = 1.5*U*_eq_(C)]. Other H atoms were placed in calculated position (methylene C—H = 0.95Å and aromatic C—H=0.95 Å) and refined as riding [*U*_iso_(H) = 1.2*U*_eq_(C)].

### Crystal data

**Table:**

C_13_H_14_Cl_2_O	*D*_x_ = 1.390 Mg m^−^^3^
*M**_r_* = 257.14	Melting point: 385 K
Orthorhombic, *P**n**m**a*	Mo *K*α radiation, λ = 0.71073 Å
Hall symbol: -P 2ac 2n	Cell parameters from 3552 reflections
*a* = 11.2553 (7) Å	θ = 2.2–27.0°
*b* = 7.0458 (4) Å	µ = 0.50 mm^−^^1^
*c* = 15.4969 (9) Å	*T* = 173 K
*V* = 1228.94 (13) Å^3^	Block, colorless
*Z* = 4	0.47 × 0.39 × 0.15 mm
*F*(000) = 536	

### Data collection

**Table:**

Bruker SMART 1000 CCD diffractometer	1444 independent reflections
Radiation source: fine-focus sealed tube	1227 reflections with *I* > 2σ(*I*)
graphite	*R*_int_ = 0.021
ω scans	θ_max_ = 27.0°, θ_min_ = 2.2°
Absorption correction: multi-scan (SADABS; Sheldrick, 2004)	*h* = −12→14
*T*_min_ = 0.798, *T*_max_ = 0.928	*k* = −8→9
6162 measured reflections	*l* = −19→14

### Refinement

**Table:**

Refinement on *F*^2^	Primary atom site location: structure-invariant direct methods
Least-squares matrix: full	Secondary atom site location: difference Fourier map
*R*[*F*^2^ > 2σ(*F*^2^)] = 0.032	Hydrogen site location: inferred from neighbouring sites
*wR*(*F*^2^) = 0.085	H-atom parameters constrained
*S* = 1.07	*w* = 1/[σ^2^(*F*_o_^2^) + (0.0391*P*)^2^ + 0.6612*P*] where *P* = (*F*_o_^2^ + 2*F*_c_^2^)/3
1444 reflections	(Δ/σ)_max_ < 0.001
95 parameters	Δρ_max_ = 0.28 e Å^−^^3^
0 restraints	Δρ_min_ = −0.36 e Å^−^^3^

### Special details

**Table:**

Geometry. All e.s.d.'s (except the e.s.d. in the dihedral angle between two l.s. planes) are estimated using the full covariance matrix. The cell e.s.d.'s are taken into account individually in the estimation of e.s.d.'s in distances, angles and torsion angles; correlations between e.s.d.'s in cell parameters are only used when they are defined by crystal symmetry. An approximate (isotropic) treatment of cell e.s.d.'s is used for estimating e.s.d.'s involving l.s. planes.
Refinement. Refinement of *F*^2^ against ALL reflections. The weighted *R*-factor *wR* and goodness of fit *S* are based on *F*^2^, conventional *R*-factors *R* are based on *F*, with *F* set to zero for negative *F*^2^. The threshold expression of *F*^2^ > σ(*F*^2^) is used only for calculating *R*-factors(gt) *etc*. and is not relevant to the choice of reflections for refinement. *R*-factors based on *F*^2^ are statistically about twice as large as those based on *F*, and *R*- factors based on ALL data will be even larger.

### Fractional atomic coordinates and isotropic or equivalent isotropic displacement parameters (Å^2^)

**Table:**

	*x*	*y*	*z*	*U*_iso_*/*U*_eq_	Occ. (<1)
Cl1	0.73794 (5)	0.2500	0.45333 (4)	0.03276 (17)	
Cl2	0.34147 (5)	0.2500	0.26347 (3)	0.03138 (17)	
C1	0.57275 (19)	0.2500	0.61286 (13)	0.0239 (4)	
H1	0.6571	0.2500	0.6149	0.029*	
C2	0.51505 (19)	0.2500	0.68766 (13)	0.0265 (5)	
H2	0.4307	0.2500	0.6869	0.032*	
C3	0.57686 (18)	0.2500	0.77240 (13)	0.0225 (4)	
C4	0.49783 (18)	0.2500	0.85260 (12)	0.0227 (4)	
C5	0.5740 (2)	0.2500	0.93380 (14)	0.0314 (5)	
H5A	0.6163	0.1518	0.9347	0.047*	0.50
H5B	0.5225	0.2500	0.9848	0.047*	
H5C	0.6252	0.3625	0.9340	0.047*	0.50
C6	0.41949 (13)	0.0709 (2)	0.85096 (10)	0.0285 (3)	
H6A	0.3670	0.0751	0.8005	0.043*	
H6B	0.3714	0.0656	0.9036	0.043*	
H6C	0.4701	−0.0421	0.8477	0.043*	
C7	0.51674 (18)	0.2500	0.52726 (13)	0.0224 (4)	
C8	0.58317 (17)	0.2500	0.45064 (13)	0.0217 (4)	
C9	0.53111 (19)	0.2500	0.36945 (13)	0.0244 (4)	
H9	0.5785	0.2500	0.3187	0.029*	
C10	0.40892 (19)	0.2500	0.36426 (13)	0.0241 (4)	
C11	0.33861 (18)	0.2500	0.43745 (15)	0.0267 (5)	
H11	0.2544	0.2500	0.4329	0.032*	
C12	0.39335 (19)	0.2500	0.51767 (14)	0.0265 (5)	
H12	0.3451	0.2500	0.5680	0.032*	
O1	0.68512 (13)	0.2500	0.77742 (10)	0.0323 (4)	

### Atomic displacement parameters (Å^2^)

**Table:**

	*U*^11^	*U*^22^	*U*^33^	*U*^12^	*U*^13^	*U*^23^
Cl1	0.0204 (3)	0.0528 (4)	0.0251 (3)	0.000	0.00297 (19)	0.000
Cl2	0.0291 (3)	0.0413 (3)	0.0237 (3)	0.000	−0.0039 (2)	0.000
C1	0.0243 (10)	0.0245 (10)	0.0228 (10)	0.000	0.0018 (8)	0.000
C2	0.0232 (10)	0.0337 (12)	0.0225 (10)	0.000	−0.0004 (8)	0.000
C3	0.0221 (10)	0.0236 (10)	0.0218 (10)	0.000	0.0009 (8)	0.000
C4	0.0202 (9)	0.0284 (11)	0.0195 (9)	0.000	0.0007 (8)	0.000
C5	0.0256 (11)	0.0463 (14)	0.0223 (10)	0.000	−0.0029 (9)	0.000
C6	0.0269 (7)	0.0303 (8)	0.0285 (8)	−0.0032 (6)	0.0043 (6)	0.0008 (6)
C7	0.0238 (10)	0.0204 (10)	0.0229 (10)	0.000	0.0011 (8)	0.000
C8	0.0190 (9)	0.0209 (10)	0.0252 (10)	0.000	0.0019 (8)	0.000
C9	0.0276 (11)	0.0240 (10)	0.0217 (10)	0.000	0.0039 (8)	0.000
C10	0.0286 (11)	0.0220 (10)	0.0217 (10)	0.000	−0.0019 (8)	0.000
C11	0.0205 (10)	0.0311 (12)	0.0285 (11)	0.000	0.0005 (8)	0.000
C12	0.0256 (11)	0.0310 (11)	0.0228 (10)	0.000	0.0055 (8)	0.000
O1	0.0200 (7)	0.0468 (10)	0.0301 (8)	0.000	0.0026 (6)	0.000

### Geometric parameters (Å, °)

**Table:**

Cl1—C8	1.742 (2)	C5—H5C	0.9800
Cl2—C10	1.737 (2)	C6—H6A	0.9800
C1—C2	1.329 (3)	C6—H6B	0.9800
C1—C7	1.469 (3)	C6—H6C	0.9800
C1—H1	0.9500	C7—C12	1.397 (3)
C2—C3	1.486 (3)	C7—C8	1.403 (3)
C2—H2	0.9500	C8—C9	1.388 (3)
C3—O1	1.221 (2)	C9—C10	1.378 (3)
C3—C4	1.528 (3)	C9—H9	0.9500
C4—C5	1.522 (3)	C10—C11	1.383 (3)
C4—C6	1.5398 (19)	C11—C12	1.387 (3)
C4—C6^i^	1.5398 (19)	C11—H11	0.9500
C5—H5A	0.8400	C12—H12	0.9500
C5—H5B	0.9799		
			
C2—C1—C7	125.3 (2)	H6A—C6—H6B	109.5
C2—C1—H1	117.3	C4—C6—H6C	109.5
C7—C1—H1	117.3	H6A—C6—H6C	109.5
C1—C2—C3	122.83 (19)	H6B—C6—H6C	109.5
C1—C2—H2	118.6	C12—C7—C8	116.09 (18)
C3—C2—H2	118.6	C12—C7—C1	121.53 (19)
O1—C3—C2	121.57 (19)	C8—C7—C1	122.39 (18)
O1—C3—C4	121.93 (18)	C9—C8—C7	122.84 (19)
C2—C3—C4	116.50 (17)	C9—C8—Cl1	116.34 (15)
C5—C4—C3	110.15 (17)	C7—C8—Cl1	120.82 (15)
C5—C4—C6	109.64 (11)	C10—C9—C8	118.32 (19)
C3—C4—C6	108.65 (11)	C10—C9—H9	120.8
C5—C4—C6^i^	109.64 (11)	C8—C9—H9	120.8
C3—C4—C6^i^	108.65 (11)	C9—C10—C11	121.55 (19)
C6—C4—C6^i^	110.09 (17)	C9—C10—Cl2	119.27 (16)
C4—C5—H5A	109.5	C11—C10—Cl2	119.17 (16)
C4—C5—H5B	109.5	C10—C11—C12	118.73 (19)
H5A—C5—H5B	108.7	C10—C11—H11	120.6
C4—C5—H5C	109.4	C12—C11—H11	120.6
H5A—C5—H5C	109.5	C11—C12—C7	122.5 (2)
H5B—C5—H5C	110.2	C11—C12—H12	118.8
C4—C6—H6A	109.5	C7—C12—H12	118.8
C4—C6—H6B	109.5		
			
C7—C1—C2—C3	180.0	C1—C7—C8—C9	180.0
C1—C2—C3—O1	0.0	C12—C7—C8—Cl1	180.0
C1—C2—C3—C4	180.0	C1—C7—C8—Cl1	0.0
O1—C3—C4—C5	0.0	C7—C8—C9—C10	0.0
C2—C3—C4—C5	180.0	Cl1—C8—C9—C10	180.0
O1—C3—C4—C6	−120.11 (11)	C8—C9—C10—C11	0.0
C2—C3—C4—C6	59.89 (11)	C8—C9—C10—Cl2	180.0
O1—C3—C4—C6^i^	120.11 (11)	C9—C10—C11—C12	0.0
C2—C3—C4—C6^i^	−59.89 (11)	Cl2—C10—C11—C12	180.0
C2—C1—C7—C12	0.0	C10—C11—C12—C7	0.0
C2—C1—C7—C8	180.0	C8—C7—C12—C11	0.0
C12—C7—C8—C9	0.0	C1—C7—C12—C11	180.0

Symmetry codes: (i) *x*, −*y*+1/2, *z*.
